# Books Reviewed

**Published:** 1869-05

**Authors:** 


### Books Reviewed.

Practical Treatise on the Diseases of Women. By T. Gillard Thomas, M. D., Professor of Obstetrics and the Diseases of Women and Children in the College of Physicians and Surgeons, New York. Philadelphia: Henry C. Lea, 1869.

It is but a few months since we had occasion to notice the first appearance of this work, and to speak of the second edition in detail, would be mainly to repeat what we then said of its contents and character. It has now been before the medical public about one year, and has everywhere been received with marked approval. No department of medicine has made greater advances within the past few years, than the one to which this work is devoted, and a collected, systematic record of this progress is indispensable both to the student and practitioner.

The work is written in a concise and practical manner, yet sufficiently comprehensive to bring the subjects fully up to the recent improvements. The work has been revised in some parts, and additions have been made which add to its value. We have no space to devote to a review of any of the chapters, but regard it as quite sufficient for our present purpose when we announce the appearance of the second edition of a work, now well known to the profession. The illustrations are very numerous, well chosen, and finely executed, greatly increasing its value as a text-book. The publisher is fully entitled to thanks of the profession for the elegant and substantial manner in which his part of the work has been done. This is especially true at the present time when many valuable books are published so cheaply as to make it hardly possible to call them "bound volumes."

Nature and Time in the Cure of Diseases. Massachusetts Medical Society Prize Essay, awarded to James F. Hibberd, M. D. Boston: David Clapp & Son.

This essay fully merits the prize of the Massachusetts Medical Society, and shows that finally the physicians of that ancient and honorable State are ready to acknowledge in some degree, the influence of *nature* and *time* in the cure of disease. Years ago Dr. Bigelow of Boston, wrote truly of "Nature in Disease," and annually, ever since, the suggestion has been made to the Society that *nature* and *time* were, after all, the great curative agents. It is but a few years since the Society was stirred and its indignation aroused by the sentiment that "if all physic was thrown in the sea it would be a great deal better for mankind, and worse for the fishes," but it has now given its prize to an essay which shows that time and nature are the restoring forces in disease, inclosing, however, the following in a parenthesis: "It is understood that the Society is not to be considered as approving the doctrines contained in this dissertation." It is, perhaps, well enough to withhold approval, as a Society, but we venture that every sensible, honest member, half educated in his profession, approves the doctrines and conclusions. If not now, they all will, give them a little more time. The following is the summary:

SUMMARY.—The prominent points presented in the foregoing dissertation may be enumerated as follows:

1. All vitalized matter is the subject of a law of development peculiar to its class.

2. Vital organizations are not active *per se*, but are endowed with a capability of activity under stimulation.

3. Normal stimulants produce physiological activity or health; abnormal stimulants produce pathological activity or disease.

4. Human maladies are always the result of abnormal stimulants acting on the histological elements of the body.

5. Disease in any part continues as long as the pathological stimulant is operative; when this ceases, the part returns to its physiological state.

6. To cure disease it is only necessary to remove the stimulant exciting it.

7. This stimulant is rarely known, and still more rarely can it be removed.

8. In most diseases we only recognize the grosser symptoms, after the initial processes have completed their course.

9. After the stage of recognition most diseases must pursue their course through a series of phenomena under an inexorable biological law.

10. The duty of the physician is to watch nature and assist her as opportunity may offer.

11. All perturbating medicines are themselves pathological stimulants, and should not be administered except under a certainty of abating a greater evil.

12. The present popular professional estimate of the medical virtues of drugs rests, mainly, on the vicious logic of *post hoc ergo propter hoc*.

13. That this estimate is erroneous, is proven by:

a.—Curative diseases are recovered from in the absence of all kinds of drugs.

b.—Curable diseases are recovered from under the most diverse treatment.

c.—The adulteration of drugs makes their strength uncertain.

d.—The state of the patient's mind makes the operation of even pure drugs uncertain.

14. A recognition of the doctrine of the *vis medicatrix naturæ* must underlie all rational therapeutics.

15. The principle involved in this phrase has been recognized and deferred to, since the earliest historical era of medicine, and is likely to be immortal.

16. It derogates nothing from the physician, or the agents he uses, that nature is predominant, and art opiferous.

